# Correlation-Based Network Analysis of the Influence of *Bemisia tabaci* Feeding on Photosynthesis and Foliar Sugar and Starch Composition in Soybean

**DOI:** 10.3390/insects13010056

**Published:** 2022-01-05

**Authors:** Inana X. Schutze, Pedro T. Yamamoto, José B. Malaquias, Matthew Herritt, Alison Thompson, Paul Merten, Steve E. Naranjo

**Affiliations:** 1 Department of Entomology and Acarology, Luiz de Queiroz College of Agriculture, University of São Paulo, Piracicaba 13400-000, Brazil; pedro.yamamoto@usp.br; 2 Institute of Biosciences, São Paulo State University, São Paulo 44540-000, Brazil; malaquias.josebruno@gmail.com; 3 Arid-Land Agricultural Research Center, U.S. Department of Agriculture, Maricopa, AZ 85138, USA; matthew.herritt@usda.gov (M.H.); alison.thompson@usda.gov (A.T.); paul.merten@usda.gov (P.M.); steve.naranjo@usda.gov (S.E.N.)

**Keywords:** sweetpotato whitefly, *Glycine max*, turn-over number, fructose, chlorophyll

## Abstract

**Simple Summary:**

*Bemisia tabaci* affects plant performance by feeding directly from its energy sources, making it difficult to quantify crop damage, which is indirectly assessed through losses in productivity. The goal of this study was to characterize the influence of *B. tabaci* feeding on soybean, an economically valuable crop, and attempt to identify the optimal parameter for direct quantification of crop damage. A correlation network was created to extract biological interactions between the plant and *B. tabaci* nymphs at different densities and plant stages. Nymphs were more abundant during the vegetative stage, and a strong correlation between the density of nymphs and starch and fructose content was observed. The photosynthetic parameter turn-over number N was positively correlated with nymph density at a low-infestation level and negatively correlated with nymphs when they occur at a high infestation level. This association between nymph density and N may allow for development of a ranking scale to predict pest density, representing a useful tool for evaluating the potential impact of *B. tabaci* on soybean, especially in large areas, where nymph monitoring can be time-consuming.

**Abstract:**

*Bemisia tabaci* (MEAM1) represents a species of economic importance in soybean. One of the obstacles to the management of *B. tabaci* is the quantification of damage by the pest because damage is indirectly inferred through losses in productivity. The objective of this study was to characterize the influence of *B. tabaci* feeding on soybean by assessing effects on photosynthetic parameters and the sugar and starch content of soybean leaves. The goal was to identify the optimal parameter to directly quantify pest damage on crop yield. Correlation networks were created among data on sugar content (fructose, glucose, and sucrose), starch and photosynthetic parameters (initial fluorescence, performance index on absorption basis, and turn-over number), and the number of nymphs at each of three infestations level (low, medium, and high) during both the vegetative and reproductive stage of the crop. In general, nymphs were more abundant during the vegetative stage. Starch content was strongly correlated with nymph density. A strong positive correlation was observed between fructose and nymph density during the vegetative stage. Among the photosynthetic parameters, the turn-over number N was positively correlated with nymph density at a low-infestation level and negatively correlated with nymphs when they occurred at a high-infestation level. *B. tabaci* feeding affected the plant’s physiology and its interaction is reflected in part by the relationships among photosynthetic parameters as well as the levels of sugars and starch. This understanding might be useful in developing better monitoring tools for pest management.

## 1. Introduction

The whitefly, *Bemisia tabaci* MEAM1 (Gennadius 1889) (Hemiptera: Aleyrodidae), is considered one of the most destructive invasive pests of many crops, including soybean (*Glycine max* (L.) Merril) [1]. Whiteflies were considered a secondary pest in soybean until the early 2000s, but today, it represents a species of economic importance [2], responsible for causing direct damage through the removal of phloem sap and indirectly favoring formation and growth of the fungus (*Capnodium* sp.) that causes sooty mold due to the excretion of honeydew.

One of the obstacles to the management of *B. tabaci* is the ability to quantify the damage caused by the pest, as these are indirectly inferred through losses in productivity and are invariably confounded by the impacts of other pests during the soybean production cycle. An alternative to directly assessing the damage caused by *B. tabaci* is to estimate the effects of its feeding on photosynthesis and the sugar and starch composition of the leaves.

The interaction between the chemical composition of leaves and the performance of whiteflies has been investigated in eggplant, and results show that varieties with higher glucose and sucrose concentrations provide better conditions for the development of *B. tabaci* [3]. A positive correlation between carbohydrate levels and whitefly populations also has been reported in eggplant and other solanaceous crops [4], and higher amounts of glucose and fructose also contribute to higher densities of whiteflies in cotton [5]. In soybean, the profile of sugars in the leaves change with the phenological stage, and the presence and intensity of whitefly feeding also may affect the plant’s physiology. Sugars, such as glucose and sucrose, are associated with photosynthesis and thus yield, and they also are important to *B. tabaci* development [6,7]. These elements play a role in the development of *B. tabaci* and might be useful for understanding the influence of *B. tabaci* on soybean at different phenological stages under different pest population densities.

Another way to investigate the injury caused by *B. tabaci* in soybeans is by assessing how infestations interfere with photosynthesis. Reductions in photosynthetic rate caused by *B. tabaci* infestations has been reported for many crops, including tobacco, eggplant, zucchini, and cotton [8,9,10,11]. *B. tabaci* feeding has been associated with a reduction in the net photosynthetic rate in zucchini [10,11], and infestations has been associated with reductions in the photosynthetic rate related to decreases in chlorophyll content, photosynthetic capacity, and stomatal conductance [12]. It is known that *B. tabaci* causes damage to soybean [13], resulting in yield losses and reduction in grain weight. The objective of this study was to identify the influence of *B. tabaci* feeding on soybean by assessing effects on photosynthetic parameters, chlorophyll, and the sugar and starch content of soybean leaves, with a goal of establishing better parameters to quantify crop damage by this pest.

## 2. Materials and Methods

### 2.1. Bemisia tabaci Rearing

Whiteflies were reared in cages in a greenhouse using soybean (*Glycine max* (L.) Merril) as the host. Plants were grown in 2-L pots in a greenhouse and replaced every 20 days. The initial whitefly population was collected from a cotton field at the United States Department of Agriculture—Arid Land Agricultural Research Center (USDA-ALARC) in Maricopa, Arizona, USA, and was previously identified as *B. tabaci* biotype B (MEAM1). Molecular characterization of the insects was made periodically during the study for confirmation of the insect biotype [14].

### 2.2. Establishment of Soybean Plants

Soybean (*var.* 01057408) was sown in seed trays containing a general-purpose growing medium. When the seedlings reached the V1 phenological stage [15], they were transplanted individually to a 5-L pot containing 9 parts growing medium/5 parts sand, both sterilized. This process was repeated after two weeks to obtain soybean in two different phenological stages for further infestation with *B. tabaci*. Each plant was covered with an anti-aphid screen attached to the bottom of the pot that served as a cage for eventual whitefly infestation. The experiment was carried out under greenhouse conditions.

### 2.3. Soybean Infestation with Bemisia tabaci

For the infestation, adults of *B. tabaci* were collected from the rearing culture with an aspirator made with an 8-mm transparent plastic hose, “voil” fabric, and a 10-mL pipette tip and were placed in Eppendorf^®^ tubes and released into the cages containing the soybean plants.

Soybean at V2 and R1 phenological stages were infested at three different levels on the same date. The control treatment contained no insects (Table 1). The experimental unit was a single cage.

The experiment was carried out in a completely randomized design consisting of eight treatments with 12 replications. The experiment was repeated for a total of 24 replicates per treatment. Sampling of insects and plants ended simultaneously for both phenological stages.

### 2.4. Bemisia tabaci Population Density

A leaflet from the middle third of each plant was collected every two weeks for the duration of the experiment for a total of six samples and placed individually in a transparent plastic bag for transport to the laboratory. Samples were refrigerated until they were processed. Counts were made from the leaflet abaxial surface with a stereoscopic microscope (40× magnification). Then, the leaf area of the sample was measured with a LI-3100C (LI-COR, Inc., Lincoln, NE, USA).

### 2.5. Photosynthetic Parameters by Chlorophyll Fluorescence Induction Kinetics

For the Chlorophyll Fluoresce Induction Kinetics (OJIP) protocol, a leaf on the plant was dark-adapted for at least 20 min with detachable leaf clips. Measurements were then performed using the FluorPen FP100 (Photo System Instruments, Czech Republic).

A clip was attached to one of the leaflets in the upper middle third of the plant before the measurements. The device emits a saturation pulse through a beam of light, which when reflected, is read by the device. The OJIP protocol is a dark-adapted chlorophyll fluorescence technique used to measure plant stress. The parameters measured include initial fluorescence (F_0_), performance index on absorption basis (Pi_Abs), and turn-over number (N). These assays were performed in situ in the greenhouse weekly.

### 2.6. Sampling and Sample Preparation

The leaflets were collected every two weeks, stored individually in 50-mL centrifuge tubes, and immediately frozen in liquid nitrogen, after which they were stored in a freezer at −80 °C. One leaflet per plant was collected, totaling 12 leaflets per treatment in each evaluation. Samples were collected bi-weekly for six weeks.

The leaflets were individually homogenized in liquid nitrogen using a pestle and a mortar, weighted in two aliquots of 20 mg each sample, placed in 2-mL Eppendorf^®^ tubes, and stored in a −80 °C freezer for later analyses of chemical composition (sugars and starch) and chlorophyll (*a*, *b*, and *a* + *b*).

### 2.7. Chemical Composition Assessment of Soybean Leaves

An assessment of the quantity and quality of sugars and starch present in the infested soybean leaves was carried out for all the leaf samples previously prepared. The evaluation of the sugar content was carried out by first preparing the samples for extraction of non-structural carbohydrates and then extracting the starch for subsequent chromatographic analysis.

#### 2.7.1. Extraction of Non-Structural Carbohydrates

A total of 25 µL of maltose (20 mg/mL of 80% ethanol) and 1 mL of 80% ethanol were added to the tubes containing the samples; the tubes were placed in a water bath at 85 °C for 10 min and then centrifuged for 5 min at 10,000 rpm. The supernatant was transferred to a new 2-mL tube. Then, 0.5 mL of 80% ethanol was added to the tubes containing the precipitate, submitting them again to the water bath and centrifugation as described above two more times, adding the supernatants each time. The tubes containing the total supernatant were adjusted to 2 mL with 80% ethanol and centrifuged once more for 5 min at 10,000 rpm. The samples were then transferred to HPLC vials using a syringe and a 0.45-µm filter. The sugars assessed were fructose, glucose, and sucrose. Maltose could not be analyzed because the test protocols for other sugars required the addition of maltose, which was used as a surrogate.

#### 2.7.2. Starch Extraction

Then, 25 µL of maltose (20 mg/mL in 80% ethanol) and 500 µL of 2M potassium hydroxide were added to the tubes containing the centrifuged precipitate resulting from the extraction of non-structural carbohydrates, placed in a water bath at 100 °C for 10 min, and then allowing to cool to room temperature. Then, 100 µL of 1M acetic acid (PH = 4.5), 50 µL of Tris 1M buffer solution (PH = 7.2), and 100 µL of alpha-amylase were added. The tubes were placed in a water bath at 85 °C for 30 min and allowed to cool to room temperature. A total of 100 µL of 1M acetic acid (PH = 4.5) and 500 µL of amyl glucosidase were added, and again, tubes were placed in a water bath at 100 °C for 4 min. Finally, 75 µL of 2M sodium hydroxide was added and the volume of the tubes adjusted to 2 mL with 100% ethanol for centrifugation at 6000 rpm for 5 min. The sample solution was then transferred to HPLC vials using a syringe and a 0.45-µm filter.

#### 2.7.3. HPLC Analyses

The analyses of content were determined in 2 mL of each sample following methods proposed for measurements of nonstructural carbohydrates in plant tissues [16] using a high-performance liquid chromatography system (Shimadzu) composed of a Luna 5u NH2 100Å column with dimensions of 250 × 4.6 mm 5 µm column (Phenomenex) and a refractive index detector (Shimadzu).

### 2.8. Evaluation of Chlorophyll Content

For the extraction of chlorophyll, 1 mL of 100% methanol at 4 °C was added to the 2-mL Eppendorf^®^ tubes containing the samples previously weighted. The tubes were completely covered with aluminum foil to avoid exposure to light and placed on a shaking plate in a refrigerator at 4 °C for 48 h, inverting the tubes twice a day to keep the samples homogeneous. After 48 h, the samples were centrifuged at 10,000 rpm for 10 min, then 200 µL of the supernatants were transferred to propylene microplates to perform the readings.

The chlorophyll contents were obtained by spectrophotometry at absorbances 652 and 665, performed with the Synergy^®^ HT microplate reader (BioTek, Winooski, VT, USA). The absorbances were then used in the calculations of chlorophyll *a*, *b*, and *a* + *b* by multiplying the absorbance values by the respective correction factor (A_665.2_ = 0.478) or (A_652_ = 0.466) divided by the sample weight (mg) to obtain the value in µg/mg using the following equations (Porra et al., 1989):

Chl *a* (μg/mL) = 16.29 A_665.2_ − 8.54 A_652_

Chl *b* (μg/mL) = 30.66 A_652_ − 13.58 A_665.2_

Chl *a* + *b* (μg/mL) = 22.12 A_652_ + 2.71 A_665.2_

### 2.9. Statistical Analysis

Statistical analyses were performed using the R Core Team [17] program. Data were checked for normality, homogeneity, and suitability by the Shapiro–Wilk [18] and Bartlett [19] tests for further analysis of variance (ANOVA). Multiple comparisons of means were assessed by Tukey’s test. We used the functions shapiro.test and bartlett.test from the package stats in R Core Team [17] (2019) program to test normality and homogeneity, respectively.

In the univariate analyses, we observed no significant interactions between infestation treatment and plant growth stage for any of the variables. We also found no differences between treatments or stages (*p* > 0.05, Table S1). However, when we considered all variables in a multivariate analysis with the Permutation test using the package vegan in R Core Team [17], we observed that there was a significant effect of plant growth stage (Supplementary material).

We conducted a correlation network analysis with the package corrr in R Core Team [17] to assess correlations among variables. The graphical displays show which variables appear closer together and are joined by stronger paths. The paths were colored by their sign (red for positive and blue for negative). The proximity of the points was determined using multidimensional clustering.

Correlation networks were created from data on sugar content, starch, and photosynthetic parameters, connecting these factors to the number of nymphs at each infestation level (low, medium, and high) in two phenological stages, vegetative and reproductive. The more solid the color of the line, the stronger the correlation between the factors. Correlation coefficients and significance levels are provided in Tables S2–S4.

## 3. Results

### 3.1. Bemisia tabaci Population Density

The number of nymphs was significantly different between the treatments both in the vegetative and reproductive stages, and we observed higher numbers as infestation levels increased. Differences in leaf area were observed only during the vegetative stage (Table 2).

### 3.2. Photosynthetic Parameters, Sugars, and Starch Composition

The objective of the correlation network analysis was to observe how the interactions between the factors evaluated were altered by different densities of nymphs. Because the control treatments were not infested with *B. tabaci*, they are not included in this correlation network analysis.

**Figure 1:**
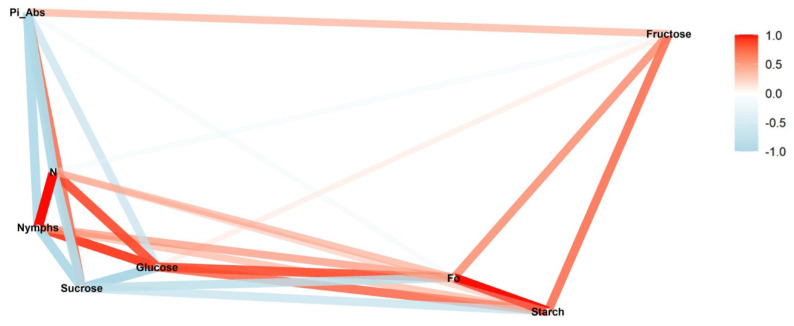
Correlation between nymphs of *Bemisia tabaci* biotype B (MEAM1), in the low-infestation treatment (T2), and photosynthetic parameters (initial fluorescence (F_0_), performance index on absorption basis (Pi_Abs), and turn-over number (N)), sugars (glucose, fructose, and sucrose), and starch during the reproductive soybean stage. The red and blue paths represent positive and negative correlations, respectively, between the variables. Statistical results are provided in Table S2.

**Figure 2:**
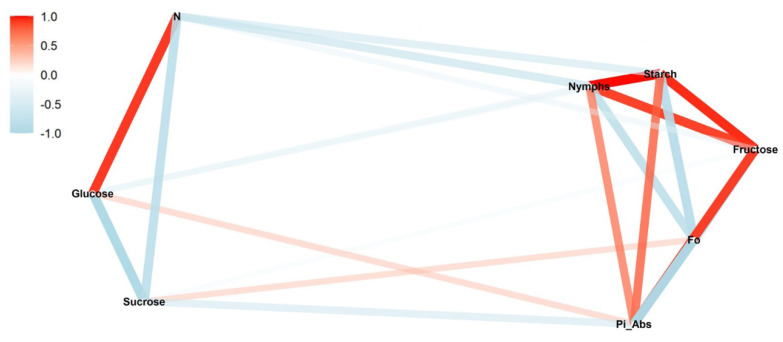
Correlation between nymphs of *Bemisia tabaci* biotype B (MEAM1) in the low-infestation treatment (T6) and photosynthetic parameters (initial fluorescence (F_0_), performance index on absorption basis (Pi_Abs), and turn-over number (N)), sugars (glucose, fructose, and sucrose), and starch during the vegetative soybean stage. The red and blue paths represent positive and negative correlations, respectively, between the variables. Statistical results are provided in Table S2.

**Figure 3:**
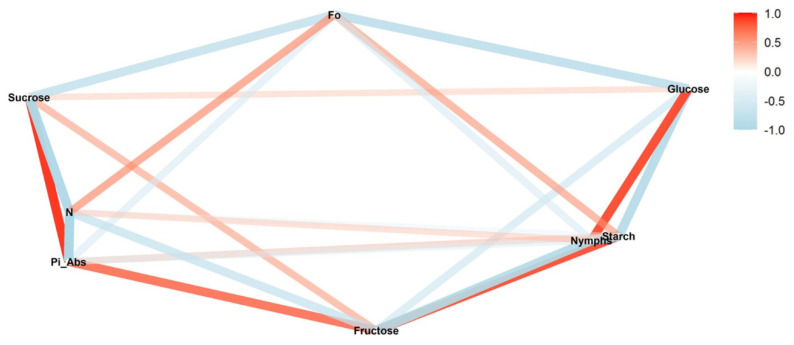
Correlation between nymphs in the medium-infestation treatments (T3) and photosynthetic parameters (initial fluorescence (F_0_), performance index on absorption basis (Pi_Abs), and turn-over number (N)), sugars (glucose, fructose, and sucrose), and starch during the reproductive soybean stage. The red and blue paths represent positive and negative correlations, respectively, between the variables. Statistical results are provided in Table S3.

**Figure 4:**
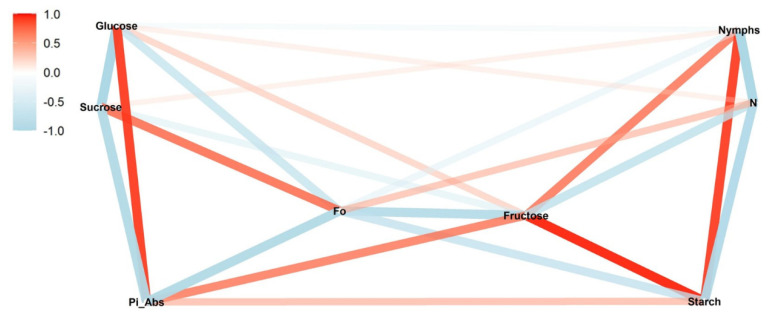
Correlation between nymphs in the medium-infestation treatments (T7) and photosynthetic parameters (initial fluorescence (F_0_), performance index on absorption basis (Pi_Abs), and turn-over number (N)), sugars (glucose, fructose, and sucrose), and starch during the vegetative soybean stage. The red and blue paths represent positive and negative correlations, respectively, between the variables. Statistical results are provided in Table S3.

**Figure 5:**
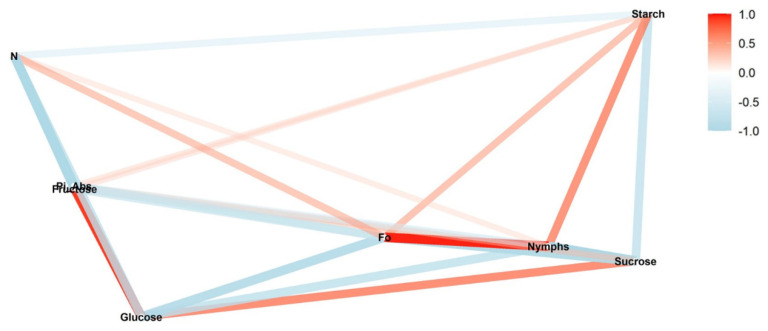
Correlation between nymphs in the high-infestation treatment (T4) and photosynthetic parameters (initial fluorescence (F_0_), performance index on absorption basis (Pi_Abs), and turn-over number (N)), sugars (glucose, fructose, and sucrose), and starch during the reproductive soybean stage. The red and blue paths represent positive and negative correlations, respectively, between the variables. Statistical results are provided in Table S4.

**Figure 6:**
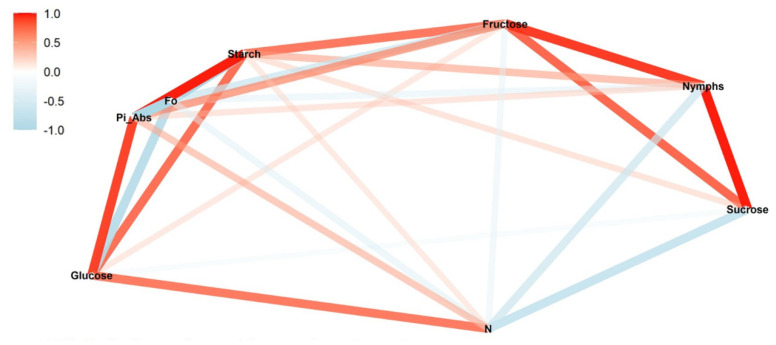
Correlation between nymphs in the high-infestation treatments (T8) and photosynthetic parameters (initial fluorescence (F_0_), performance index on absorption basis (Pi_Abs), and turn-over number (N)), sugars (glucose, fructose, and sucrose), and starch during the vegetative soybean stage. The red and blue paths represent positive and negative correlations, respectively, between the variables. Statistical results are provided in Table S4.

In the low infestation during the reproductive stage, the greatest positive correlation occurred between nymphs and turnover number N (*ρ* = 0.99; *p* < 0.01) (Figure 1). This correlation was negative for the low infestation during the vegetative stage (*ρ* = −0.67; *p* < 0.05) (Figure 2). The same inversion also was observed for all the other levels of infestation (Figure 3, Figure 4, Figure 5 and Figure 6).

In the reproductive stage, the correlation of nymphs and Pi_Abs (*ρ* = −0.92; *p* < 0.01) was negative; however, in the vegetative stage, these correlations were positive (*ρ* = −0.71; *p* < 0.01). This same pattern was observed between fructose and nymphs. The networks also show that Pi_Abs and fructose are positively correlated in all the treatments.

Another correlation observed for all the infestation levels was between F_0_ and starch. In the reproductive stage, a strong positive correlation was observed (Figure 1, Figure 3 and Figure 5); this correlation also appeared strong but negative in the vegetative stage (Figure 2, Figure 4 and Figure 6).

We observed different patterns of correlation between the reproductive and vegetative stages (Figure 1 and Figure 2). In the reproductive stage, positive correlation was observed between nymphs and glucose (*ρ* = 0.91; *p* < 0.05) (Figure 1). In the vegetative stage, the same correlation was negative but not significantly (*ρ* = −0.38; *p* > 0.05) (Figure 2).

While phenological stages altered the correlation network, the different infestation treatments also affected the relationships between the parameters in the correlation network. For medium infestation in the reproductive stage, we observed a weak negative and not significant correlation between nymphs and fructose (*ρ* = −0.16; *p* > 0.05) and a positive correlation between nymphs and starch (*ρ* = 0.48; *p* < 0.05) (Figure 3) and between nymphs and glucose (*ρ* = 0.91; *p* < 0.01).

In the vegetative stage, the correlations of nymphs with fructose (*ρ* = 0.75; *p* < 0.05) and starch (*ρ* = 0.91; *p* < 0.01) were positive (Figure 4). For the photosynthetic parameters, the correlations were mostly negative. Pi_Abs did not significantly correlate with nymphs.

In the high-infestation reproductive stage, there was a negative correlation between nymphs and glucose (*ρ* = −0.71; *p* < 0.05) and also a strong positive correlation between nymphs and starch (*ρ* = 0.70; *p* < 0.05) (Figure 5), which indicates that this might be one of the main factors responsible for increasing the density of nymphs. A similar pattern regarding nymphs and starch was observed in the treatments T3, T7, and T8, which presented a higher density of nymphs (Figure 3, Figure 4 and Figure 6). For photosynthetic parameters, F_0_ showed a positive correlation with nymphs (*ρ* = 0.97; *p* < 0.01), but again no significant correlation was observed with Pi_Abs (Figure 5).

In the high-infestation vegetative stage treatment, the correlations of nymphs with sugars were positive for fructose (*ρ* = 0.93; *p* < 0.01) and sucrose (*ρ* = 0.97; *p* < 0.01) and moderate and non-significant for starch (*ρ* = 0.52; *p* > 0.05). No significant correlation was observed for glucose (*ρ* = −0.06; *p* > 0.05) (Figure 6). For photosynthetic parameters, correlation strengths were lower; Pi_Abs was positively (*ρ* = −0.32; *p* > 0.05) and F_0_ negatively correlated (*ρ* = −0.38; *p* > 0.05), while N was again negative (*ρ* = −0.66; *p* < 0.05), following the same pattern described for the low-infestation treatment.

### 3.3. Chlorophyll Content

There were differences in the chlorophyll *a* content between plants in the vegetative and reproductive stages. A higher concentration in the vegetative stage was observed for all levels of infestation although there was no difference between treatments in the same plant phenological stage (Table 3).

Compared with the previous parameters evaluated, the difference in chlorophyll *a* content between phenological stages were similar to the changes observed for interactions between nymphs and fructose, sucrose, and Pi_Abs, which presented a positive correlation in the vegetative stage and negative in the reproductive. On the contrary, the correlation between nymphs and turn-over number was negative in the vegetative stage treatments and positive in the reproductive treatments (Figure 1, Figure 2, Figure 3, Figure 4, Figure 5 and Figure 6). Chlorophyll *b* was lowest in the high-infestation treatment during the vegetative stage and the medium-infestation during the reproductive stage (Table 4).

A similar pattern was observed in differences for chlorophyll *b* and the nymphs’ correlations between sucrose and starch. In the vegetative stage, the lower content of chlorophyll *b* in the high-infestation level was concomitant with a stronger positive correlation between nymphs and sucrose and a weaker positive correlation between nymphs and starch (Figure 6). For the reproductive stage, the lower content of chlorophyll *b* in the medium-infestation level was concomitant with a weaker negative correlation between nymphs and sucrose and a stronger negative correlation between nymphs and starch (Figure 3). An inversion also was observed in the correlation between nymphs and F_0_ in medium infestation in the reproductive stage; the correlation was positive for low and high treatments and negative in the medium infestation (Figure 3).

When analyzing chlorophyll *a* + *b*, the differences previously observed for chlorophyll *a* and *b* are presented together. Content was lowest for the high infestation during the vegetative stage and the medium infestation during the reproductive stage (Table 5). These differences were similar to those observed for sucrose and starch correlations with nymphs.

## 4. Discussion

Despite its size, *B. tabaci* may affect the plant’s physiology in a way that ultimately translates into yield loss [20]. Unlike defoliating pests, like caterpillars, which reduce the photosynthetic area of the leaves, or stinkbugs, which cause direct damage to grains, the whitefly affects plant performance by feeding directly from its energy sources.

When soybean was infested in the vegetative stage, nymphal density increased faster than with infestation in the reproductive stage. It is possible that during the vegetative stage, more nutrients are available in the leaves, as the plant is allocating photosynthate to the promotion of plant growth, consequently providing better conditions for the development of *B. tabaci*. After reaching the reproductive stage, these resources are reallocated to the production of flowers and grains [21], with fewer nutrients available in the leaves, and thus perhaps fewer nutrients available to promote *B. tabaci* growth. Although infestations occurred at the same time, the number of nymphs in the vegetative stage was more than four times higher than in the reproductive stage. This high density of nymphs (208.89 per cm²) likely affected leaf growth, as was reflected in reduced leaf area (Table 2).

Ideally, in a healthy plant, the initial fluorescence (F_0_) from a photosynthetic organ like a leaf should be lower compared with treatments that subject the plant to some type of stress [22]. Feeding by *B. tabaci* induced stress that was measurable through changes in chlorophyll fluorescence. While there was a positive correlation between nymphal density and F_0_ at both low- and high-infestation rates, there was a negative correlation for medium infestations. The same pattern of correlations was observed for starch. This is consistent with a reduction in chlorophyll *b* content in the medium infestation during the reproductive stage. Previous studies have shown that increases in chlorophyll *b* content results in increased amount of starch content, both indicators of growth vigor in plants [23,24]. Like starch, a similar pattern was observed for F_0_, which is one most representative chlorophyll fluorescence parameters and widely used in studies to measure stress on plants [25].

F_0_ also showed a correlation with starch. The correlation between these parameters was positive in the reproductive stage and negative in the vegetative stage. This pattern was observed for all infestation treatments but was strongest under low infestation. Starch is an energy reservoir, synthesized in photosynthetic cells and degraded to provide substrates for photosynthesis and sucrose for the plant [26]. We also observed a positive correlation between starch and F_0_ at higher nymph density during the vegetative stage. At the same time, the sucrose content was inversely proportional to starch. Higher concentration of starch in the leaves indicates a healthier plant, capable of providing higher levels of sucrose for plant growth, but also better conditions for *B. tabaci* development [3].

F_0_ and starch were positively correlated in all the infestation levels during the reproductive stage. Even when nymphs were negatively correlated with F_0_ and with starch at medium infestations, the positive correlation between F_0_ and starch was still observed, indicating that the initial fluorescence is influenced not only by stress but also by starch content. During the vegetative stage, a negative correlation between nymphs and F_0_ was observed at all infestation levels. However, during this stage, the correlation between F_0_ and starch was negative in all the treatments, supporting the relationship between starch and initial fluorescence.

For the absorption flow performance index (Pi_Abs), the expected response is the inverse of that expected for F_0_. When the plants are healthy and free from stress, they are expected to perform better in the absorption of light by chlorophyll pigments in the leaves. Non-stressed plants should present higher indexes of absorption, while plants subject to stress through higher insect infestations should show a negative correlation, corresponding to a lower vitality of the plant [22]. Treatments in the reproductive stage showed a negative correlation as expected. No significant correlation was observed for medium and high infestation in the vegetative stage, probably due to the high density of nymphs interfering with the absorption of light by the leaves. However, a positive correlation was observed in the low infestation at the vegetative stage. This could be explained due to the negative correlation of Pi_Abs with N. The Pi_Abs indicates light absorbance performance, while the turn-over (N) represents the conversion rate of this light energy to chemical energy. In this case, the plant would be showing a higher absorbance performance in relation to energy conversion that is occurring at a lower frequency because of nymphal feeding. Thus, the higher concentration of chlorophyll *a* in the vegetative stage results in better absorption, allowing the plant to store more energy than it is consumed (i.e., a lower conversion rate (N)).

The N, or turn-over number, represents how many times the primary quinone acceptor (Q^A^) was reduced to Q^A-^ in the interval from time zero (t_0_) to reach maximum fluorescence (F_m_) after receiving electrons from water using light energy. This direct electron transfer is essential in the process of converting light energy into chemical energy [22,27]. An increase in the absorption index would be reflected in a lower reduction rate of the quinone acceptor (Q^A^) such that the plant would be absorbing more light but not converting this light energy to photoassimilates. Previous study has shown that increases in F_0_, which is inverse to Pi_Abs, represents more Q^A-^ formation [27], i.e., the apparently higher light absorption does not mean the plant has better photosynthetic performance. In the medium-infestation treatment, N was negatively correlated with all the parameters studied except F_0_ and nymphs. Higher levels of chlorophyll (*a* + *b*) were observed in the vegetative treatments, and during the reproductive treatments, the medium infestation showed the lowest concentration. We also observed the inversion in the correlations between nymphs and starch and between nymphs and F_0_ in the medium-infestation-level treatments. In other words, besides a higher light absorption, the plant also depends on good photosynthetic performance to provide resources such as starch for plant health, making it a more suitable host to *B. tabaci*.

Whiteflies are phloem-feeders, and phloem is responsible for transporting photoassimilates, with generally high concentrations of sugars [28]. The primary sugars associated with the high occurrences of whiteflies are sucrose and glucose, responsible for improving survival and adult longevity and fecundity, and promoting feeding, oviposition preference, and immature development [3]. As these sugars are also related to photosynthesis, a negative relationship between *B. tabaci* infestation and photosystem activity is expected. Here, that situation resulted in poorer plant development, as indicated by smaller leaves in soybean under high insect infestation.

The vegetative stage appears to provide better conditions for the insect’s development, as evidenced by the faster growth of nymphs during this stage. Differing from that observed for the other sugars, the correlations between glucose with nymphs were non-significant, indicating that during the vegetative stage other components, such as fructose and starch, may provide better conditions for *B. tabaci* development. Glucose is second only to maltose as a product of starch breakdown and a source of carbon exported from chloroplasts during its degradation [29].

Even though glucose also is a product of starch breakdown, it did not present negative correlation with starch. First, it could be that maltose provided an energetic advantage over the export of glucose, representing two-thirds of the carbon export, while glucose represents the rest [29]. Secondly, because it represented a smaller part in the leaf composition, it could have been affected by a reduction in the leaflet size, especially in the high-infestation treatment. In contrast, in the high infestation, there was no significant correlation between glucose and nymphs, and that might be due to compensation by the plant that reduced the concentration of resources (glucose), therefore mitigating stress on the leaves by reducing leaf size [30].

One of the main components of honeydew excreted by whiteflies is fructose [31,32], which has also been reported for other hemipterans [33,34]. A strong positive correlation between fructose and nymphs was observed for all treatments at the vegetative stage, when the greatest number of *B. tabaci* nymphs occurred. A similar pattern was observed for sucrose. Varieties with higher content of fructose and sucrose in their leaves were reported to promote higher densities and better conditions for the development of *B. tabaci* in eggplants and cotton, respectively [3,5]. On the other hand, a positive correlation was observed between nymphs and glucose instead of fructose during the reproductive stage. This could be due to a variation in sugar availability in soybean leaves from the vegetative to reproductive stage [6]. During the vegetative stage, sucrose, which is also a precursor of fructose, is abundant in the leaves, and levels start to decrease during the reproductive stage as it is being relocated to reproductive structures [6,7]. This pattern was observed for all except the high-infestation level, where a positive correlation occurred between nymphs and starch. Nonetheless, glucose is still available via starch breakdown, supplying *B. tabaci* needs during the reproductive stage.

Among the photosynthetic parameters, the turn-over number N was positively correlated with nymphs at lower densities of nymphs and negatively correlated with nymphs at higher densities. These correlations also were affected by the phenological stage, whereby correlations were negative during the vegetative and positive during the reproductive stage. Nevertheless, this pattern is modified somewhat by the density of infestation. During the reproductive stage, the correlation is strongest at the lowest density of infestation and declines in strength with increasing density. During the vegetative stage, the negative correlation between nymphs and N is weakest at the lowest infestation and grows in strength as density increases at the medium infestation. Although the maximum correlation was not observed at the high infestation compared with the medium infestation, a strong negative correlation was still observed. This may have been influenced by the significant drop in chlorophyll content observed at the high infestation affecting N. The assessment of the correlation between nymphs and N may allow for development of a ranking scale of N values to predict density of nymphs and might represent a useful tool for evaluating the impact of *B. tabaci* in the crop, especially in large areas, where nymph monitoring can be time consuming.

*B. tabaci* feeding affects the plant’s physiology reflected in part by the relationships among photosynthetic parameters as well as the levels of sugars and starch. From our results, the greatest impacts of insect infestation occur during the vegetative phenological stage, when plants are more suitable for *B. tabaci* development, thus leading to plant stress that will likely have consequences on productivity.

## Figures and Tables

**Table 1:** Infestation levels of *Bemisia tabaci* released in cages containing soybean in phenological stages V2 and R1 and the expected population density (nymphs per leaflet) in each treatment.

Treatment	Infestation Level	Number of *B. tabaci* Adults Released	Soybean Phenological Stage at Infestation	Expected Population Density (Nymphs/Leaflet)
T1	Control	-	R1	0
T2	Low	20	R1	40
T3	Medium	40	R1	120
T4	High	80	R1	240
T5	Control	-	V2	0
T6	Low	20	V2	40
T7	Medium	40	V2	120
T8	High	80	V2	240

**Table 2:** Average number of nymphs of *Bemisia tabaci* biotype B (MEAM1) per leaflet and leaflet area at each infestation level for the vegetative stage (V_2_) and reproductive stage (R_2_) of soybean.

Infestation Levels	V_2_		R_2_	
	Nymphs	Area (cm²)	Nymphs	Area (cm²)
Control	0.00 ± 13.87 ^a^	38.43 ± 1.31 ^a^	0.00 ± 3.78 ^a^	40.38 ± 0.91 ^ns^
Low	56.14 ± 13.87 ^ab^	37.92 ± 1.39 ^a^	12.75 ± 3.78 ^ab^	38.93 ± 0.99
Medium	90.53 ± 13.87 ^b^	34.96 ± 1.41 ^ab^	19.88 ± 3.78 ^b^	38.49 ± 0.96
High	208.89 ± 13.87 ^c^	32.97 ± 1.47 ^b^	46.50 ± 3.78 ^c^	38.14 ± 0.99

Within each column values followed by a different letter are significantly different (*p* < 0.05; Tukey’s test).

**Table 3:** Chlorophyll *a* (µg/mg) content in soybean leaflets at each *Bemisia tabaci* infestation level during the vegetative and reproductive stages.

Infestation Levels	Vegetative	Reproductive
Control	3.53 ± 0.14 ^aA^	2.93 ± 0.16 ^aB^
Low	3.64 ± 0.14 ^aA^	2.85 ± 0.16 ^aB^
Medium	3.50 ± 0.14 ^aA^	2.73 ± 0.16 ^aB^
High	3.52 ± 0.14 ^aA^	3.01 ± 0.16 ^aB^

Values in a row followed by the different upper-case letters and in a column followed by different lower-case letters are significantly (*p* < 0.05) different (Tukey’s test). Mean value ± standard error.

**Table 4:** Chlorophyll *b* (µg/mg) content in soybean leaflets at each *Bemisia tabaci* infestation level for the vegetative and reproductive stages.

Infestation Levels	Vegetative	Reproductive
Control	2.50 ± 0.18 ^aA^	2.08 ± 0.23 ^aA^
Low	2.26 ± 0.18 ^aA^	2.07 ± 0.23 ^aA^
Medium	2.57 ± 0.18 ^aA^	1.76 ± 0.23 ^bA^
High	1.91 ± 0.18 ^bA^	2.13 ± 0.23 ^aA^

Values in a row followed by the different upper-case letters and in a column followed by the different lower-case letters are significantly (*p* < 0.05) different (Tukey’s test). Mean value ± standard error.

**Table 5:** Chlorophyll *a* + *b* (µg/mg) content in soybean leaflets at each *Bemisia tabaci* infestation level for the vegetative and reproductive stages.

Infestation Levels	Vegetative	Reproductive
Control	6.04 ± 0.24 ^aA^	5.01 ± 0.29 ^aB^
Low	5.92 ± 0.24 ^aA^	4.91 ± 0.29 ^aB^
Medium	6.08 ± 0.24 ^aA^	4.49 ± 0.29 ^bB^
High	5.43 ± 0.24 ^bA^	5.14 ± 0.29 ^aB^

Values in a row followed by the different upper-case letters and in a column followed by the different lower-case letters are significantly (*p* < 0.05) different (Tukey’s test). Mean value ± standard error.

## Data Availability

Not applicable.

## Supplementary Materials

The following supporting information can be downloaded at: https://www.mdpi.com/article/10.3390/insects13010056/s1, Table S1: Means ± SE for various parameters for the main effects of insect infestation level and plant growth stage, Table S2: Matrix of correlation coefficients between nymphs of Bemisia tabaci biotype B (MEAM1), and photosynthetic and sugar content parameters for the low infestation treatment, Table S3: Matrix of correlation coefficients between nymphs of *Bemisia tabaci* biotype B (MEAM1), and photosynthetic and sugar content parameters for the medium infestation treatment, Table S4: Matrix of correlation coefficients between nymphs of *Bemisia tabaci* biotype B (MEAM1), and photosynthetic and sugar content parameters for the high infestation treatment.
