# Supplementary material for: Correlation-Based Network Analysis of the Influence of Bemisia tabaci Feeding on Photosynthesis and Foliar Sugar and Starch Composition in Soybean
Source: Insects. 2022 Jan 5;13(1):56. doi: 10.3390/insects13010056 (PMC8779753; doi:10.3390/insects13010056)
Supplement: Supplementary file 1 [file insects-13-00056-s001.zip › insects-1491399-supplementary.pdf]

insects

Supplementary Material

Statistical analysis

All statistical analyses were performed using the R Core Team (2019) program [1]. A two-way ANOVA was used to test for differences between infestation treatments and plant growth stage. We checked for normality and homogeneity of variance. When necessary the data were transformed using the Box-Cox (1964) method using the function boxcox from MASS package [2]. In addition, we used a multivariate analysis with a Permutation test with the package vegan [3].

Results

In the univariate analyses we observed no significant interaction between treatment and stage for F_0 ($F_{3,24} = 0.16$; $P = 0.91$); fructose ($F_{3,24} = 0.15$; $P = 0.93$); glucose ($F_{3,24} = 0.92$; $P = 0.44$); N ($F_{3,24} = 1.22$; $P = 0.32$); Pi_Abs ($F_{3,24} = 0.04$; $P = 0.98$); starch ($F_{3,24} = 0.38$; $P = 0.76$) and sucrose ($F_{3,24} = 0.21$; $P = 0.88$). We also observed no difference between the treatments or stages for these variables ($P > 0.05$). However, when we considered all variables in the multivariate analysis with Permutation test, we observed significant plant growth stage effects ($F = 34.239$; $P = 0.001$).

Table S1. Means ± SE for various parameters for the main effects of insect infestation level and plant growth stage.

**Treatment**	**Fructose/Treatment**	**Glucose/Treatment**	**Starch/Treatment**	**Sucrose/Treatment**	**Fo/Treatment**	**Pi_Abs/Treatment**	**N/Treatment**
Control	0.0016±0.0001	0.0003±0.00010	0.2692±0.0074	0.0009±0.00007	7088.81±199.78	5.27±0.60	750.84±27.42
Low	0.0016±0.0002	0.0002±0.00004	0.2626±0.0069	0.0009±0.00007	6971.07±211.56	5.33±0.66	723.52±31.88
Medium	0.0018±0.0002	0.0002±0.00006	0.2637±0.0084	0.0009±0.00007	7116.50±182.24	5.16±0.53	835.00±85.51
High	0.0018±0.0002	0.0003±0.00005	0.2688±0.0057	0.00012±0.00018	7101.02±206.13	5.05±0.70	748.36±25.35
Stage	Fructose/Stage	Glucose/Stage	Starch/Stage	Sucrose/Stage	Fo/Stage	Pi_Abs/Stage	N/Stage
Vegetative	0.0017±0.00008	0.0003±0.00002	0.2671±0.0051	0.0009±0.00009	7168.88±156.79	5.44±0.34	744.86±17.64
Reproductive	0.0017±0.00020	0.0002±0.00006	0.2650±0.0047	0.0010±0.00005	6969.81±109.34	4.88±0.53	784.00±46.29

There was no difference between the treatments or stages by the F test ($P > 0.05$).

Table S2. - Matrix of correlation coefficients between nymphs of *Bemisia tabaci* biotype B (MEAM1), and photosynthetic and sugar content parameters for the low infestation treatment

Vegetative Stage								
	Fructose	Glucose	Starch	Sucrose	Nymphs	Fo	Pi_Abs	N
Fructose	1.00	-	-	-	-	-	-	-
Glucose	0.00^{ns}	1.00	-	-	-	-	-	-
Starch	0.96**	-0.27^{ns}	1.00	-	-	-	-	-
Sucrose	-0.20^{ns}	-0.98**	0.08^{ns}	1.00	-	-	-	-
Nymphs	0.92**	-0.38^{ns}	0.99**	0.19^{ns}	1.00	-	-	-
Fo	-0.98**	-0.18^{ns}	-0.90**	0.37^{ns}	-0.84**	1.00	-	-
Pi_Abs	0.93**	0.38^{ns}	0.79**	-0.55*	0.71**	-0.98**	1.00	-
N	-0.33^{ns}	0.94**	-0.58*	-0.86**	-0.67*	0.17^{ns}	0.04^{ns}	1.00
Reproductive Stage								
	Fructose	Glucose	Starch	Sucrose	Nymphs	Fo	Pi_Abs	N
Fructose	1.00	-	-	-	-	-	-	-
Glucose	0.23^{ns}	1.00	-	-	-	-	-	-
Starch	0.78*	0.79*	1.00	-	-	-	-	-
Sucrose	-0.12^{ns}	-0.99**	-0.71*	1.00	-	-	-	-
Nymphs	-0.16^{ns}	0.91**	0.48^{ns}	-0.95**	1.00	-	-	-
Fo	0.68*	0.86*	0.98**	-0.80*	0.60^{ns}	1.00	-	-
Pi_Abs	0.52*	-0.70*	-0.12^{ns}	0.78*	-0.92**	-0.26^{ns}	1.00	-
N	-0.26^{ns}	0.87**	0.39^{ns}	-0.92**	0.99**	0.51^{ns}	-0.96**	1.00

^{ns}= not statistically significant; *= significant (p-value between 0.01 to 0.05); **= very significant correlation coefficient (p-value < 0.01). Nymphs= number of nymphs of *B. tabaci.* F_0 = initial fluorescence. Pi_Abs = performance index on absorption basis. N= turn-over number.

Table S3. - Matrix of correlation coefficients between nymphs of *Bemisia tabaci* biotype B (MEAM1), and photosynthetic and sugar content parameters for the medium infestation treatment

Vegetative Stage								
	Fructose	Glucose	Starch	Sucrose	Nymphs	Fo	Pi_Abs	N
Fructose	1.00	-	-	-	-	-	-	-
Glucose	0.40ns	1.00	-	-	-	-	-	-
Starch	0.95	0.12ns	1.00	-	-	-	-	-
Sucrose	-0.46ns	-0.99**	-0.18ns	1.00	-	-	-	-
Nymphs	0.75*	-0.29ns	0.91**	0.22ns	1.00	-	-	-
Fo	-0.91**	-0.73*	-0.76*	0.78*	-0.43ns	1.00	-	-
Pi_Abs	0.74*	0.91**	0.51ns	-0.94**	0.11ns	-0.94**	1.00	-
N	-0.79*	0.23ns	-0.93**	-0.16ns	-0.99**	0.48ns	-0.17ns	1.00
**Reproductive Stage**								
	Fructose	Glucose	Starch	Sucrose	Nymphs	Fo	Pi_Abs	N
Fructose	1.00	-	-	-	-	-	-	-
Glucose	0.23ns	1.00	-	-	-	-	-	-
Starch	0.78*	0.79*	1.00	-	-	-	-	-
Sucrose	-0.12ns	-0.99**	-0.71*	1.00	-	-	-	-
Nymphs	-0.16ns	0.91**	0.48*	-0.95**	1.00	-	-	-
Fo	0.68*	0.86*	0.99**	-0.80**	0.60ns	1.00	-	-
Pi_Abs	0.52*	-0.70*	-0.12ns	0.78*	-0.92**	-0.26 ns	1.00	-
N	-0.26ns	0.87**	0.39	-0.92**	0.99**	0.51ns	-0.96**	1.00

ns= not statistically significant; *= significant (p-value between 0.01 to 0.05); **= very significant correlation coefficient (p-value < 0.01). Nymphs= number of nymphs of *B. tabaci*. F_0 = initial fluorescence. Pi_Abs = performance index on absorption basis. N= turn-over number.

Table S4.- Matrix of correlation coefficients between nymphs of *Bemisia tabaci* biotype B (MEAM1), and photosynthetic and sugar content parameters for the high infestation treatment

	Fructose	Glucose	Starch	Sucrose	Nymphs	Fo	Pi_Abs	N
**Vegetative Stage**								
Fructose	1.00	-	-	-	-	-	-	-
Glucose	0.30^{ns}	1.00	-	-	-	-	-	-
Starch	0.79*	0.81	1.00	-	-	-	-	-
Sucrose	0.84*	-0.26^{ns}	0.33^{ns}	1.00	-	-	-	-
Nymphs	0.93**	-0.06^{ns}	0.52^{ns}	0.97**	1.00	-	-	-
Fo	-0.69*	-0.89**	-0.98**	-0.19^{ns}	-0.38^{ns}	1.00	-	-
Pi_Abs	0.64^{ns}	0.92**	0.97**	0.12^{ns}	0.32^{ns}	-0.99**	1.00	-
N	-0.35^{ns}	0.78*	0.28^{ns}	-0.80*	-0.66*	-0.43^{ns}	0.48^{ns}	1.00
**Reproductive Stage**								
	Fructose	Glucose	Starch	Sucrose	Nymphs	Fo	Pi_Abs	N
Fructose	1.00	-	-	-	-	-	-	-
Glucose	0.91**	1.00	-	-	-	-	-	-
Starch	0.28^{ns}	-0.12^{ns}	1.00	-	-	-	-	-
Sucrose	0.38^{ns}	0.72*	-0.77*	1.00	-	-	-	-
Nymphs	-0.47^{ns}	-0.79*	0.70*	-0.99**	1.00	-	-	-
Fo	-0.67*	-0.91**	0.51^{ns}	-0.94**	0.97**	1.00	-	-
Pi_Abs	1.00**	0.91**	0.29^{ns}	0.38^{ns}	-0.47^{ns}	-0.67*	1.00	-
N	-0.97**	-0.80**	-0.49^{ns}	-0.17^{ns}	0.27^{ns}	0.49^{ns}	-0.97**	1.00

^{ns}= not statistically significant; *= significant (p-value between 0.01 to 0.05); **= very significant correlation coefficient (p-value < 0.01). Nymphs= number of nymphs of *B. tabaci*. F_0 = initial fluorescence. Pi_Abs = performance index on absorption basis. N= turn-over number.

References

1. *R Core Team. R: A Language and Environment for Statistical Computing;* **2021**, *R Foundation for Statistical Computing: Vienna, Austria*
2. Venables, W.N.; Ripley, B.D. *Modern Applied Statistics with S*, 4th ed.; Statistics and Computing; Springer: New York, NY, USA, 2002; ISBN 978-1-4419-3008-8.
3. Oksanen, J.; Blanchet, F.G.; Friendly, M.; Kindt, R.; Mcglinn, D.; Minchin, P.R.; Hara, R.B.O.; Simpson, G.L.; Stevens, M.H.H.; Szoecs, E.; et al. vegan: Community Ecology Package. R package version 2.5-6. 2019. http://CRAN.R-project.org/package=vegan Accessed on March 23, 2021.
